# Striatal transcriptomic alterations immediately after short-term abstinence from methamphetamine self-administration in rats

**DOI:** 10.1186/s13041-025-01249-z

**Published:** 2025-11-04

**Authors:** Won-Jun Jang, Sang-Hoon Song, Taekwon Son, In Soo Ryu, Jung Hoon Jung, Sooyeun Lee, Chul-Ho Jeong

**Affiliations:** 1 College of Pharmacy, Keimyung University, 1095 Dalgubeol-Daero, Dalseo-Gu, Daegu, 42601 Republic of Korea; 2 Korea Brain Bank, Korea Brain Research Institute, Daegu, 41062 Republic of Korea; 3 Biorchestra Co., Ltd., Daejeon, 34013 Republic of Korea

**Keywords:** Drug addiction, Methamphetamine, Biomarkers, Key genes, Reward mechanisms, Synaptic plasticity

## Abstract

Substance use disorders, particularly drug addiction, are complex neurophysiological conditions characterized by cycles of compulsive drug use, withdrawal symptoms, and relapses. Methamphetamine (MA) addiction evolves through repeated exposure, altering brain circuits related to reward and neuroplasticity. The need for reliable biomarkers to diagnose and monitor MA addiction has become increasingly critical in clinical practice. In this study, we explored the time-dependent transcriptomic changes in the rat striatum immediately after short-term abstinence following MA self-administration. Using a rat model, we conducted RNA sequencing to analyze the transcriptomic alterations in the striatum immediately after the self-administration and short-term abstinence phases (12- and 24-h post-MA). Through protein–protein interaction (PPI) network analysis and gene expression pattern assessment, we identified key genes that demonstrated significant expression changes. These genes were strongly linked to reward mechanisms, synaptic plasticity, and memory processes, suggesting a role in mediating MA-associated behaviors. Understanding the expression dynamics of these genes provides valuable insights into the molecular mechanisms underlying MA addiction and offers a foundation for developing diagnostic tools and therapeutic strategies targeting addiction-related neural adaptations.

**Supplementary Information:**

The online version contains supplementary material available at 10.1186/s13041-025-01249-z.

## Introduction

Methamphetamine (MA) is a highly addictive psychostimulant that induces physical and psychological dependence by disrupting the central nervous system (CNS). The initial use of MA induces powerful euphoria and arousal effects, which promote drug-seeking behavior, and repeated drug use subsequently reinforces addiction [1, 2]. From a neurophysiological perspective, drug addiction is characterized by an abnormal increase in dopamine release, resulting in excessive activation of the brain’s reward circuitry [3, 4]. This excessive activation is accompanied by changes in the density of neurotransmitter receptors, resulting in changes in drug sensitivity [5, 6]. These physiological changes cause the brain’s reward system to be hyperactivated during drug use, thereby producing intense pleasure. However, severe withdrawal symptoms manifest upon cessation of drug use [7, 8]. These withdrawal symptoms are major factors contributing to relapse after periods of abstinence [9, 10].

Short-term abstinence is a critical period during which significant metabolic and neurological adjustments occur following MA use [2, 11]. During this period, the body initiates recovery from the drug’s effects, although the process often involves an imbalance in neurotransmitter systems and the emergence of withdrawal symptoms [2, 8, 12]. Withdrawal symptoms refer to the physical and psychological responses that occur when drug use is reduced or discontinued, reflecting physical dependence or addiction. Drug withdrawal typically begins within a few hours of the last dose and serves as a key marker of dependence rather than a direct indicator of addiction, which involves compulsive drug use and behavioral impairments [8, 13]. Short-term abstinence provides an opportunity to observe immediate and dynamic changes in brain function and gene expression associated with drug cessation [12, 14, 15]. Understanding these changes is essential for identifying the biological mechanisms underlying reward, withdrawal, and the potential transition to addiction.

Recent studies have further highlighted the importance of region-specific transcriptomic responses to MA exposure. Miao et al. (2023) demonstrated that MA administration induces distinct transcriptomic and epigenetic changes across multiple brain regions in male rats, emphasizing the localized nature of MA-induced molecular adaptations in the brain [16]. In parallel, Daiwile et al. (2024) showed that incubation of MA craving in punishment-resistant rats was associated with the activation of specific gene networks in the dorsal striatum, suggesting molecular signatures underlying compulsive drug-seeking behavior [17].

Based on these findings, our study focuses on dynamic transcriptomic changes in the striatum during short-term abstinence following MA self-administration. Unlike previous studies that emphasized chronic exposure or compulsivity, our work highlights gene expression dynamics during the early abstinence phase, a critical but underexplored period in the addiction trajectory. This approach provides novel insights into the early molecular adaptations occurring after cessation of MA intake and offers a valuable foundation for identifying candidate biomarkers relevant to withdrawal. These insights could inform treatment strategies and support long-term recovery.

To investigate the molecular effects of MA immediately after the short-term abstinence phase, we used an MA self-administration model in male rats. We analyzed transcriptomic changes in the striatum, focusing on transcripts with significant expression changes during MA intake and at 12 and 24 h of short-term abstinence following cessation. Additionally, we constructed a PPI network to identify key genes within the transcriptomic dataset. Through Comparative Toxicogenomics Database (CTD)-enriched disease analysis of these transcripts, we identified 10 potential key genes, analyzed their stage-specific expression patterns, and investigated their molecular functions at the transcriptomic level.

Building on these findings, we analyzed the dynamic expression patterns of key transcripts identified immediately after the MA self-administration and short-term abstinence phases. By analyzing these patterns, we sought to interpret their biological significance in the context of MA-induced neuroplasticity and reward processing. This approach aims to provide critical insights into the molecular mechanisms underlying MA use and withdrawal, ultimately contributing to the development of diagnostic biomarkers and potential therapeutic targets for MA use disorders.

## Methods

### Animals

Adult male Sprague–Dawley (SD) rats weighing 310–350 g (Daehan Animal, Seoul, Republic of Korea) were housed individually in the experimental animal facility. The environmental conditions were consistently maintained at a temperature of 22 ± 2 °C and humidity of 60 ± 2%, under a 12-h light–dark cycle (lights on at 7:00 AM). Prior to the experiment, the rats were acclimated to the housing facility and provided ad libitum access to food and water. The experimental protocol was approved by the Institutional Animal Care and Use Committee of the Korea Institute of Toxicology, Daejeon, Republic of Korea (Approval No. 1702–0076) and adhered to the institutional guidelines for ethical scientific research. All methods involving animals are reported in accordance with ARRIVE guidelines. Euthanasia was done at the end of the study using carbon dioxide in accordance with the scientific research guidelines and regulations of the Korea Institute of Toxicology.

### MA self-administration and short-term abstinence model

MA self-administration and short-term abstinence were performed as described previously [11], with the addition of detailed information on reagents and apparatus used in the present study. Following acclimation, 8-week-old rats were trained in operant conditioning chambers equipped with two levers (active and inactive) and an infusion pump. Food training was conducted using 45 mg food pellets during daily 1-h sessions to establish lever-pressing behavior. Only rats that obtained more than 80 pellets/day for 3 consecutive days were selected for surgery. Indwelling jugular catheters (inner diameter 0.02″, outer diameter 0.03″; Dow Corning, Midland, MI, USA) were implanted under pentobarbital anesthesia (50 mg/kg, i.p.) and secured with Mersilene surgical mesh (Ethicon Inc., Somerville, NJ, USA). The catheter was exteriorized via a skin incision in the back and connected to a 22-gauge stainless steel cannula (Plastics One, Roanoke, VA, USA) fixed to the head assembly with dental cement. Catheter patency was maintained by daily flushing with 0.2 mL of sterile saline containing heparin (30 IU/mL) and gentamicin sulfate (0.33 mg/mL). After a recovery period of at least 5 days, the rats self-administered MA (0.05 mg/kg per infusion) (M, n = 24) or saline (S, n = 8) under a fixed-ratio 1 (FR-1) schedule with a 20-s timeout for 2 h per day over a 16-day period. In the S group (n = 8) and M group (n = 24), only animals showing stable lever-pressing behavior with less than 20% variation over the last three days of self-administration were included in subsequent experiments. Based on this criterion, 5 rats from the S group and 16 rats from the M group were selected. The M group was further divided into the M group (n = 4), MA12 group (n = 6), and MA24 group (n = 6). Four rats from each group were randomly selected for RNA sequencing analysis. The collected striatal tissue samples from each rat were immediately frozen in liquid nitrogen after dissection at each experimental stage and stored at -80 °C until analysis. After completion of all animal experiments, total RNA was extracted from all frozen striatal samples of all groups in a single batch to minimize potential batch-to-batch variation in RNA quality and downstream sequencing results.

### RNA extraction and RNA sequencing

Total RNA was extracted from whole striatal samples using the standard TRIzol® reagent method (Invitrogen, Carlsbad, CA, USA). The quality and concentration of the RNA were measured using a filter-based multimode microplate reader (FLUOstar Omega, BMG Labtech, Ortenberg, Germany), followed by quality control (QC) analysis to assess suitability for RNA sequencing. For RNA sequencing, four samples from each group were randomly selected from those that passed the QC. The selected total RNA was prepared for mRNA sequencing library construction using the TruSeq RNA Sample Preparation Kit v2 (Illumina, San Diego, CA, USA) according to the manufacturer’s protocol. Briefly, 1 μg of total RNA was used to isolate mRNA via poly-A capture with RNA purification beads, followed by enzymatic fragmentation. First- and second-strand cDNA synthesis was then performed, after which A-tailing and end repair were performed to ligate proprietary primers. Unique sequencing adapters with indexes were incorporated to enable identification of Illumina reads from multiplexed samples in a single sequencing lane. The samples were sequenced on an Illumina HiSeq 2000 platform with paired-end 101 bp reads using the TruSeq SBS Kit v3 (Illumina, San Diego, CA, USA). Raw imaging data were converted into sequence data through base-calling and stored in FASTQ format. The paired-end reads from sixteen independent samples were trimmed for both PCR and sequencing adapters using Cutadapt. The trimmed reads were aligned to the rn6 rat reference genome using STAR (version 2.7.0e) [18], and gene-level read counts were generated using the featureCounts function from the Subread package (version 1.6.4) [19].

### Statistical analysis of differentially expressed genes (DEGs)

To identify DEGs, we analyzed the raw count data obtained from RNA sequencing using the NetworkAnalyst web tool (https://www.networkanalyst.ca/). Briefly, the data were normalized using the Trimmed Mean of M-values (TMM) method, and significant features were selected based on a false discovery rate (FDR) of less than 0.05 using edgeR [20, 21]. DEG analysis between groups was conducted by comparing the saline self-administration group (S) with the MA self-administration group (M), the 12-h abstinence group after MA self-administration (MA12), and the 24-h abstinence group after MA self-administration (MA24). Gene expression differences between the self-administration groups were analyzed based on the criteria of FDR < 0.05 and a fold change (FC) of ≥ 1.5. The DEGs identified between the groups through differential expression analysis were further refined using Venn diagram analysis to select the final set of DEGs. The expression patterns of the selected DEGs were visualized using hierarchical clustering and a heatmap. For clustering, the complete method was applied, and the distance was calculated using the Euclidean method.

### Addiction-related genes (ARGs) list

We selected two reports [22, 23] to compile a list of ARGs. The ARG list used in this study was constructed based on the human ARG dataset reported by Li et al. (PLoS Comput Biol, 2008) and the rat-specific ARG dataset established by Song et al. (Sci Rep, 2018). Li et al. (2008) systematically collected 2,343 independent pieces of evidence from more than 1,000 peer-reviewed publications published between 1976 and 2006, integrating findings from multiple experimental platforms, including single-gene studies, transcriptomic/proteomic profiling, QTL mapping, and genetic linkage and association studies, to build the Knowledgebase for Addiction-Related Genes (KARG). Our previous study, Song et al. (2018) adapted and validated this KARG dataset to generate a rat-specific ARG list applicable to experimental addiction models. In the present study, these two resources were integrated and deduplicated to construct the final ARG dataset. The ARGs identified in these reports were converted to their corresponding rat gene homologs, and overlapping genes between the reports were consolidated, yielding a total of 957 ARGs.

### PPI network construction and network centrality analysis

The PPI network was constructed using the STRING application in the Cytoscape web tool (https://cytoscape.org), employing the STRING 400 option for enhanced network connectivity [24, 25]. To identify hub genes within the constructed network, we used a network analysis tool in Cytoscape to calculate the betweenness centrality (BC), closeness centrality (CC), and degree centrality (DC) [24, 25]. DEGs with values above the network average for BC, CC, and DC were selected. Hub genes were determined by identifying the common genes from the BC, CC, and DC lists using a Venn diagram. In the PPI network, hub genes were marked with red nodes, while ARGs were represented by nodes with black borders. To validate the importance of the key genes, we calculated the average BC, CC, and DC values for 317 nodes. This included 60 ARGs, 50 hub genes, and 20 key genes within the network. The results are presented as a bar graph showing the centrality metrics for each selected transcript.

### Comparative toxicogenomics database (CTD)-enriched disease analysis for key genes

To analyze the functions of the 20 key genes, we used the CTD (https://ctdbase.org/), applying a p-value threshold of 0.01 and limiting the disease category to mental disorders. Disease categories enriched by the 20 key genes were displayed in a hierarchical path. The significantly enriched key genes and their associated diseases were visualized as a disease-gene network using the Cytoscape software. Additionally, 11 key genes included in the disease-gene network were represented in a heatmap according to different phases, with the normalized expression levels of the transcripts displayed in a corresponding chart. Statistical significance was assessed using one-way analysis of variance (ANOVA), followed by Bonferroni’s multiple comparisons test.

### Pattern analysis of key genes

The final 10 key genes were categorized into three distinct patterns across the S-M-MA12-MA24 phases. Additionally, the expression levels and patterns of these genes and diseases were mapped onto a disease-gene network, highlighting their relationships at specific time points: the MA self-administration, 12-h abstinence, and 24-h abstinence phases.

### Statistical analysis

Data were analyzed using one-way ANOVA and two-way ANOVA, followed by Bonferroni’s multiple comparison test using GraphPad Prism 8 (GraphPad Software, San Diego, CA, USA). All data are presented as mean ± standard error of the mean (SEM). Statistical significance was set at *p* < 0.05.

## Results

### MA self-administration and short-term abstinence

**Fig. 1:**
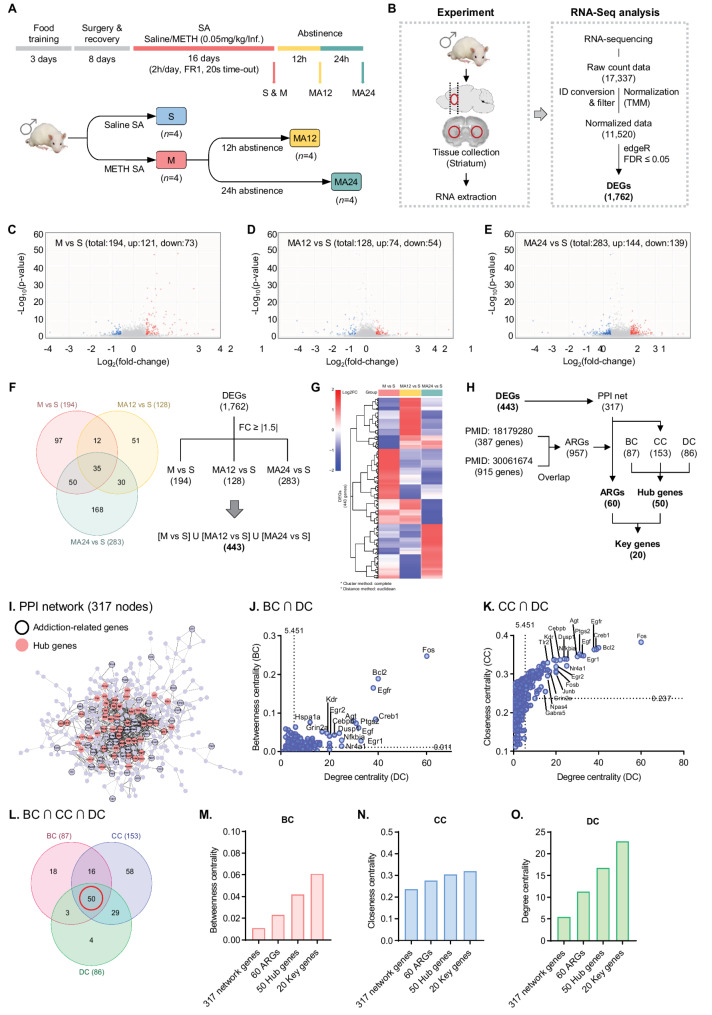
RNA-Seq analysis and identification of key genes in striatum of rats with MA self-administration and short-term abstinence. **A** Schematic overview of the animal experiment in this study. **B** Flowchart of the RNA sequencing analysis. **C**–**F** Volcano plot of DEGs: The x-axis represents the log2 FC, while the y-axis represents the -log10(p-value), reflecting the significance level of those changes. Red: up-regulated genes; blue: down-regulated genes; gray: unchanged genes in each group. **F** Venn diagram of the three groups and workflow for 443 DEGs, which are the union of three groups. **G** Heatmap and hierarchical clustering of the expression profiles of 443 DEGs. It uses hierarchical clustering with the Euclidean distance method (complete method) to generate the hierarchical tree. **H** The flowchart of identification of key genes for MA self-administration and short-term abstinence. **I** PPI network constructed using 443 DEGs. Black border: ARGs (n = 60); red node: hub gene (n = 50). **J** Scatter-plot of the betweenness centrality vs. degree centrality for 317 nodes of PPI network. **K** Scatter-plot of the closeness centrality vs. degree centrality for 317 nodes of the PPI network. The dotted lines represent the averages of BC, CC, and DC, respectively. **L** Venn diagram of BC (n = 87), CC (n = 153), DC (n = 86) groups. **M–O** Bar graphs of the mean BC, CC, and DC for 317 network genes, 60 ARGs, 50 Hub genes, and 20 key genes

Building on our previous work showing metabolomic changes immediately after short-term abstinence in MA self-administering rats [11], we now explore the transcriptomic alterations under the same conditions. MA was self-administered following the experimental schedule outlined in Fig. 1A [11]. Behavioral validation of MA self-administration was confirmed by lever-press counts and the number of infusions (Fig. S1). To analyze the transcriptomic expression patterns immediately after MA self-administration and short-term abstinence, the rats were further maintained for 12 h (MA12, n = 4) or 24 h (MA24, n = 4) after the final MA self-administration session. No significant differences were observed in the number of drug infusions among the M, MA12, and MA24 groups of MA self-administering rats (p > 0.05)(Fig. S1B).

### Gene expression profiling and identification of key genes using RNA-Seq analysis

To analyze the temporal transcriptome expression patterns immediately after MA self-administration and short-term abstinence, RNAs were isolated from the striatum, and total RNA sequencing was performed (Fig. 1B). The unique mapping rate averaged 91.52%, with raw count data identifying 17,337 genes compared to the rat reference genome (rn6). Using NetworkAnalyst, raw count data containing 17,337 features were matched at a rate of 95%, yielding 16,516 genes. Low-abundance features with raw count values of ≤ 4 and those with a variance percentile rank below 15 [26, 27], indicating unstable expression across conditions, were filtered out, leaving 11,520 genes. The data were normalized using the TMM method, and DEGs were identified using edgeR, yielding 1,762 DEGs with an FDR of ≤ 0.05 (Fig. 1B, Table S1). DEGs with an absolute FC ≥|1.5| were considered significant when comparing the M, MA12, and MA24 groups to the S group. In the M vs. S comparison, 194 DEGs were identified (121 upregulated and 73 downregulated; Fig. 1C, Table S2). For MA12 vs. S, 128 DEGs were identified (74 upregulated and 54 downregulated; Fig. 1D, Table S3). In the MA24 vs. S comparison, 283 DEGs were identified (144 upregulated and 139 downregulated; Fig. 1E, Table S4). A Venn diagram analysis was performed to compare the three groups, with 443 DEGs selected as the union across all groups (Fig. 1F, Table S5). The expression levels (FC) of the selected 443 DEGs were examined using hierarchical clustering and a heatmap. Clustering was performed using the complete cluster and Euclidean distance methods (Fig. 1G).

One potential concern with our analytical approach is that direct comparisons between the S group and the MA12 or MA24 groups are confounded by differences in both drug exposure and sampling time points, limiting the interpretability of these contrasts. To better characterize the temporal dynamics of MA-associated gene expression, we therefore conducted additional analyses comparing the M group with the MA12 and MA24 groups, as well as MA12 with MA24. Using the M group as the reference, we identified 592 DEGs, approximately 355 of which overlapped with the 443 DEGs from our original analysis. Specifically, 352 DEGs were identified in MA12 vs. M (up: 203, down: 149); 252 DEGs in MA24 vs. M (up: 81, down: 171); and 318 DEGs in MA24 vs. MA12 (up: 98, down: 220) (Fig. S2). Despite the inherent limitation of our original framework—which used the S group as the baseline and thus involved two varying factors (drug exposure and time point)—this alternative approach yielded conclusions broadly consistent with those derived from our initial design.

To identify the key genes showing dynamic expression changes immediately after MA self-administration and short-term abstinence, we performed a comparative analysis with ARGs and conducted a centrality analysis of the PPI network. The process of selecting key genes is illustrated in Fig. 1H. First, to compare the network genes with ARGs, we compiled an ARGs list. Using a PubMed search, we selected two papers [22, 23], from which we extracted 387 and 915 genes, respectively, and consolidated the overlapping genes to produce a final list of 957 ARGs (Fig. 1H, Table S6). Next, we constructed a PPI network from the 443 DEGs using the Cytoscape software, with the string 400 option applied. Networks consisting of single genes or networks composed of five or fewer genes were excluded. The largest remaining network consisted of 317 nodes and 864 edges (Fig. 1I, Table S7). To identify hub genes within the network, we performed a centrality analysis using BC, CC, and degree centrality (DC), which are commonly used measures of network centrality. Centrality analysis was conducted using the network analysis tool, Cytoscape. As a result, 87 genes for BC, 153 genes for CC, and 86 genes for DC were identified as above the network average (Table S8). The top 30 genes for each centrality measure are presented in Table S9. Additionally, the overlap between BC and DC, as well as between CC and DC, is shown in Fig. 1J and K. Notably, four genes—*Fos*, *Bcl2*, *Egfr*, and *Creb1*—ranked in the top four positions for all three centrality measures (BC, CC, and DC), confirming them as top hub genes in both the BC ∩ DC and CC ∩ DC analyses. A Venn diagram analysis of the BC ∩ CC ∩ DC overlap yielded 50 hub genes (Fig. 1L), which are indicated as red nodes in the 317-node network (Fig. 1I, Table S10).

Within the 317-node network, we identified 60 ARGs, which are marked with black-bordered nodes (Fig. 1H), and these ARGs are listed in Table S11. We examined the intersection between the 60 ARGs and 50 hub genes and ultimately identified 20 key genes. These key genes are listed in Table S10 and marked with black circles in the ARGs category. Through comparative analysis with ARGs and centrality analysis, we identified PPI network genes (317), ARGs (60), hub genes (50), and key genes (20). The average BC, CC, and DC values for the transcripts in each group were calculated and presented in a graph, demonstrating that the 20 key genes had the highest average BC, CC, and DC values. This suggests that these 20 key genes are likely to be important transcripts that were significantly altered immediately after MA self-administration and short-term abstinence (Figs. 1M–O).

### CTD disease enrichment analysis of key genes

**Fig. 2:**
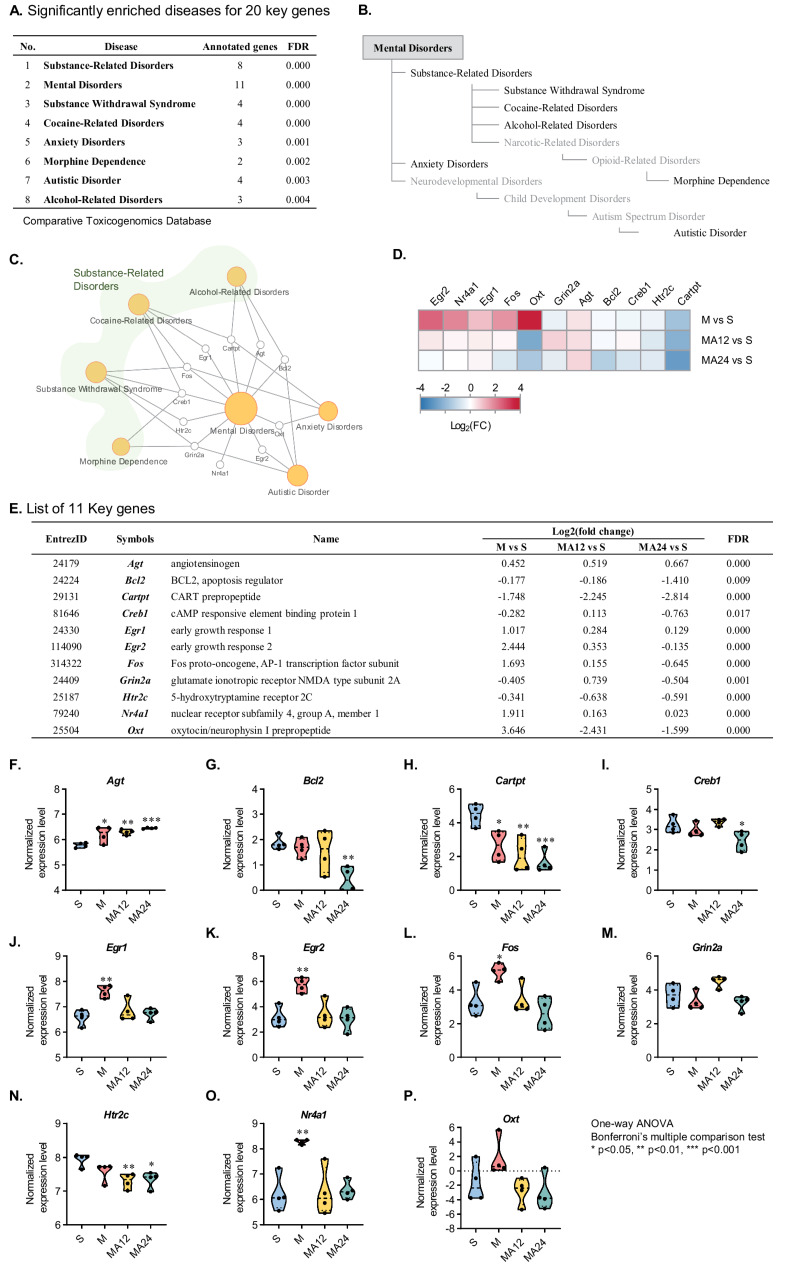
Significantly enriched disease analysis for key genes of MA self-administration and short-term abstinence. **A** List of significantly enriched diseases for 20 key genes. **B** Hierarchical paths of CTD MEDIC disease for significantly enriched diseases. **C** Disease-gene network for 11 key genes and 7 diseases. **D** Heatmap of 11 key genes for three groups (M vs. S, MA12 vs. S, MA24 vs. S). **E** List of 11 key genes for MA-self administration and short-term abstinence. **F**–**P** Normalized expression levels of 11 key genes in each phase (S-M-MA12-MA24)

The 20 key genes were identified as being significantly altered immediately after MA self-administration and short-term abstinence, indicating their potential central roles in these processes. To explore the relationship between these genes and mental disorders, we conducted an association analysis using the CTD, focusing on mental disorders that are highly relevant to addiction. The analysis revealed significant enrichment for eight diseases: substance-related disorders, mental disorders, substance withdrawal syndrome, cocaine-related disorders, anxiety disorders, morphine dependence, autistic disorder, and alcohol-related disorders (Fig. 2A). When these eight diseases were mapped onto the hierarchical paths of the CTD’s MEDIC disease classifications, they were represented as shown in Fig. 2B. Mental disorders were the top-level category, with four enriched diseases classified under substance-related disorders.

The significantly enriched diseases were broadly classified into three categories: substance-related, anxiety, and autistic disorders. A disease-gene network illustrating the relationships between the eight diseases and the enriched genes is shown in Fig. 2C. A total of 11 genes (*Egr1*, *Egr2*, *Nr4a1*, *Fos*, *Oxt*, *Grin2a*, *Agt*, *Bcl2*, *Creb1*, *Htr2c*, and *Cartpt*) were found to be involved in this network. Changes in the expression levels of these 11 genes across different phases (M, MA12, and MA24) were visualized using a heatmap (Fig. 2D). The 11 genes are listed in Fig. 2E, and their phase-specific normalized expression levels are depicted in Fig. 2F-2P. Although all 11 genes passed the thresholds of FDR < 0.05 and FC >|1.5|, one-way ANOVA with Bonferroni’s multiple comparison tests revealed that *Grin2a* and *Oxt* did not show any phase-specific expression changes. We used functional enrichment and gene-pathway analyses to examine common pathways and functional gene modules among the 11 genes (Fig. S3). We confirmed that these genes share pathways related to the regulation of neurotransmission and addiction—amphetamine addiction, cholinergic synapse, dopaminergic synapse, the cAMP signaling pathway, circadian entrainment, and cortisol synthesis and secretion (Fig. S3A)—and that the pathways and genes interact to form functional modules (Fig. S3B).

### Analysis of gene expression patterns in key genes

**Fig. 3:**
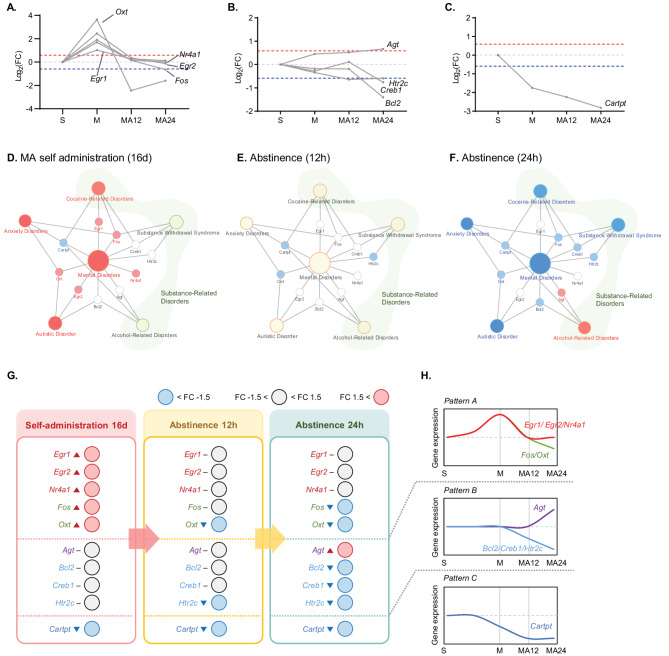
Expression pattern analysis of 10 key genes for MA self-administration and short-term abstinence. **A** Changes in Log2FC based on S phase of the five genes included in Pattern A. **B** Changes in Log2FC based on S phase of the five genes included in Pattern B. **C** Changes in Log2FC based on S phase of the one gene included in Pattern C. **D**–**F** Disease-gene up-down network for 11 key genes and 7 diseases in MA self-administration (16d), abstinence (12h), and abstinence (24h) phases. Red: up-regulated genes and diseases; blue: down-regulated genes and diseases; white: unchanged genes and diseases in each phase. **G** Diagram of expression levels for 10 key genes in each phase. **H** Simple diagram of gene expression changes of 10 key genes in each phase

We observed that the 11 identified genes generally demonstrated consistent gene expression patterns across phases. However, while 10 of these genes exhibited meaningful patterns, the expression changes in *Grin2a* were difficult to interpret. The 10 genes were categorized into three distinct expression patterns: (1) Pattern A: Genes that increased in expression during the M phase and then returned to or decreased towards the S phase levels as the experiment progressed through the MA12 and MA24 phases (Fig. 3A). This pattern included *Egr1*, *Egr2*, *Nr4a1*, *Fos*, and *Oxt*. (2) Pattern B: Genes that exhibited no significant changes during the M or MA12 phases but increased or decreased as the experiment moved into the MA12 or MA24 phases (Fig. 3B). This pattern included *Agt*, *Bcl2*, *Creb1*, and *Htr2c*. (3) Pattern C: Genes that decreased during the M phase and exhibited further declines through the MA12 and MA24 phases (Fig. 3C). *Cartpt* was the only gene with this pattern.

These 10 genes were linked to 7 enriched diseases. A disease-gene network was constructed to connect the 7 diseases and 10 genes, classifying the diseases under the broader category of mental disorders, which was further subdivided into 3 disease groups. For each phase, color coding was used to indicate whether the genes were upregulated or downregulated. When more than 50% of the genes associated with a disease exhibited similar expression trends, the disease itself was also color-coded (Fig. 3D–F).

In the M phase, we observed upregulation in four disease classes: mental disorders, cocaine-related disorders, anxiety disorders, and autistic disorder. *Egr1*, *Egr2*, *Fos*, *Nr4a1*, and *Oxt* were significantly upregulated, whereas *Cartpt* was significantly downregulated (Fig. 3D). In the MA12 phase, the previously upregulated transcripts returned to the levels observed in the saline self-administration phase. *Oxt* exhibited a decreasing trend, and *Cartpt* continued its downward expression. *Htr2c* also showed a decreased expression. The genes associated with the diseases did not exhibit more than 50% concordance in expression direction; therefore, no consistent changes were observed at the disease level (Fig. 3E). In the MA24 phase, *Egr1*, *Egr2*, and *Nr4a1* maintained the expression levels observed in the saline self-administration phase. Notably, *Cartpt* exhibited decreased expression throughout the M, MA12, and MA24 phases. *Agt*, which showed no change in the M or MA12 phases, exhibited increased expression in the MA24 phase (Fig. 3F). We then categorized the expression levels of key genes for each phase and presented them in a simplified visual format (Fig. 3G). And the three dynamic patterns of the 10 key genes immediately after MA self-administration and short-term abstinence are summarized in Fig. 3H.

## Discussion

Given recent advancements in neuroscience and the growing prevalence of drug addiction, the need for objective biological markers for diagnosing and treating MA use disorder has become increasingly urgent [25, 28, 29]. Reports underscore this urgency, indicating that approximately 61% of individuals treated for MA use disorder relapse within one year [30]. Understanding the physiological and molecular mechanisms underlying MA addiction is critical for developing effective therapeutic strategies. MA significantly alters gene expression in specific brain regions, which in turn impacts behavior through interactions within neural circuits. For instance, MA disrupts dopamine signaling pathways, leading to various addiction-related behavioral changes [31]. Therefore, studying the genetic and molecular mechanisms underlying addiction is essential for advancing research and developing targeted treatments.

Due to ethical constraints in human studies, animal self-administration models remain essential for investigating the behavioral and neurobiological mechanisms of MA addiction [24, 28, 32, 33]. This approach enables controlled examination of drug-taking, withdrawal, and relapse, providing valuable insights into the molecular underpinnings of addictive behaviors. In this study, we employed a short-access self-administration model to investigate the reward process, a critical initial phase in the development of MA addiction. During this period, we observed dynamic transcriptomic changes in the striatum, a brain region crucial for the reward and addiction processes. The striatum plays a central role in mediating reward, motivation, reinforcement learning, and habitual drug-seeking behavior, and is strongly engaged by psychostimulants such as MA via mesolimbic and nigrostriatal dopamine systems [8, 34]. Repeated MA exposure induces long-lasting synaptic remodeling and transcriptional changes in the striatum, which are key mechanisms underlying the persistence and pathophysiology of addictive behaviors [12, 35].

Recent studies have highlighted the importance of the mechanisms underlying drug-taking and drug-seeking behavior, reward experience, self-administration memory, and extinction memory in the context of addiction. For instance, Gribkova et al. demonstrated how reward experiences are modulated throughout the addiction cycle using computational simulations [36], while others have studied the evolution of exploratory behavior, self-administration memory, and extinction memory over time [37]. These aspects are likely interconnected with the behavioral changes observed during withdrawal, with alterations in gene expression playing a pivotal role in shaping these processes.

Through transcriptomic analysis, we identified 10 key genes in the striatum that exhibited significant expression changes immediately after the short-term abstinence phase. Functional categorization of these genes revealed three distinct expression patterns. Pattern A (Fig. 3H) included five genes—*Egr1*, *Egr2*, *Nr4a1*, *Fos*, and *Oxt*—which showed increased expression during M phase but either returned to baseline or decreased during MA12 and MA24 phases. Pattern A genes—*Egr1*, *Egr2*, *Nr4a1*, *Fos*, and *Oxt*—may have been altered due to multiple factors, including acute activation of the dopamine-MAPK/ERK-CREB pathway immediately after MA exposure [12, 14, 38], high levels of lever-pressing behavior during self-administration [37, 39], stress and arousal responses in the early withdrawal phase [2, 8], and homeostatic reorganization of transcriptional networks [34].

Immediate early genes (IEGs) are crucial regulators of synaptic plasticity and neuronal activity in the brain, playing essential roles in neuroadaptation [16, 40]. Acute MA administration significantly increases the expression of IEGs, such as *Egr-1* and *c-fos* in the brain, contributing to synaptic plasticity and modulating monoamine neurotransmitters, such as dopamine and serotonin [38]. In contrast, chronic MA exposure decreases the expression of IEGs, including *Egr*-2 and *Fos*, suggesting their involvement in long-term neuroadaptive changes linked to drug addiction [41]. This dynamic expression of IEGs warrant further validation through cell type-specific and time course-dependent transcriptomic analyses in future studies. MA also induces a pronounced increase in *Fos* expression in reward-related brain regions, such as the striatum and nucleus accumbens. This suggests that *Fos* may reinforce drug-seeking behavior and modulate reward circuits, further contributing to the addiction process [14, 39, 42].

*Nr4a1* (Nuclear receptor subfamily 4, group A, member 1, or Nur77) has recently been implicated in addiction, particularly cocaine use [43]. Overexpression of *Nr4a1* in neurons projecting from the ventral pallidum to the mediodorsal thalamus (MDT) enhances drug-seeking behaviors and drug-induced reinstatement in mice. Conversely, downregulating *Nr4a1* suppresses drug self-administration and cocaine-mediated behaviors, highlighting its critical role in regulating drug seeking, relapse, and addiction-related adaptations. Thus, *Nr4a1* is a promising target for therapeutic interventions in addiction [43].

Oxytocin (*Oxt*)**,** a hormone secreted by the posterior pituitary gland, plays a role in parturition, lactation, and social bonding. Additionally, it modulates social and reward behaviors, suggesting its potential influence on the social and emotional dimensions of drug addiction. Additionally, *Oxt* has been proposed as a therapeutic target for reducing drug-seeking behaviors and facilitating recovery [44, 45].

In our study, five genes—*Egr1*, *Egr2*, *Nr4a1*, *Fos*, and *Oxt*—exhibited increased expression immediately after MA self-administration, followed by a reduction during short-term abstinence. These patterns closely resemble the behavioral dynamics of drug-taking and drug-seeking behaviors observed in animal models. Alterations in the expression of these genes may reflect neuroplastic changes in the brain’s reward circuitry, influencing MA reward mechanisms, drug-seeking behaviors, and consumption patterns. As the abstinence period progressed, gene expression trends varied. *Egr1*, *Egr2*, and *Nr4a1* returned to the expression levels observed in the S group or decreased further during the abstinence phases (MA12 and MA24). Notably, *Oxt* expression was significantly reduced at MA12, while *Fos* and *Oxt* expression declined further at MA24. These observations underscore the potential roles of these genes in mediating the neuroplastic adaptations and behavioral shifts associated with MA withdrawal and recovery. Additionally, four of the five genes in Pattern A (*Egr1*, *Egr2*, *Nr4a1*, and *Fos*) exhibited the same MA-induced upregulation pattern as in our previous work [24], supporting their potential as molecular markers of MA responsiveness. In contrast, *Oxt* showed an opposite expression change, which may reflect time-dependent effects of short-term abstinence on gene regulation. These findings highlight the importance of considering temporal dynamics when interpreting transcriptomic alterations related to addiction.

Next, we classified genes that exhibited no significant change in expression following MA self-administration compared to the saline control but showed significant expression changes during the abstinence period as Pattern B (Fig. 3H). The genes included in Pattern B are *Agt*, *Bcl2*, *Creb1*, and *Htr2c*. *Htr2c* began to decrease after 12 h of abstinence, while *Bcl2* and *Creb1* showed a reduction after 24 h of abstinence. Additionally, *Agt* exhibited a significant increase in expression after 24 h of abstinence.

*Htr2c*, a serotonin receptor, has been implicated drug addiction, playing a key role in regulating serotonin signaling within the CNS, which in turn influences the reward system and modulates susceptibility to addictive substances, including cocaine, alcohol, and MA [46–48]. Although *Htr2c*’s role in addiction and reward mechanisms is unclear, a recent pilot study suggested that its agonist reduced self-reported alcohol and amphetamine-type substance use and cravings in participants with alcohol use disorder and MA use disorder, respectively [48]. In our study, the observed decrease in *Htr2c* expression immediately after short-term abstinence suggests that it plays a critical role in modulating the reward response to MA.

*Creb1*, which demonstrated a significant decrease in expression after 24 h of abstinence, plays a well-known role in neuronal plasticity, decision-making, and long-term memory [49, 50]. Additionally, the cAMP response element binding protein (CREB) has been implicated in the development of drug dependence, which contributes to the negative emotional state observed during the early stages of withdrawal from drug exposure [12]. In the MA self-administration model, an increase in the expression of genes linked to the CREB signaling pathway and neuroinflammation was observed [51]. Furthermore, inhibiting CREB-related signaling reduced the reward effects during MA self-administration [52]. This suggests that changes in the expression of *Creb1* could be crucial for modulating the reward system.

Pattern C (Fig. 3H) included genes that exhibited decreased expression following the M phase and maintained reduced levels throughout both the MA12 and MA24 phases. Among these genes is *Cartpt*, which encodes the cocaine- and amphetamine-regulated transcript (CART), a neuropeptide that is activated in the brain during cocaine and amphetamine use. CART plays a critical role in appetite regulation, weight maintenance [53, 54], energy balance [55], and stress responses [56]. In the context of addiction, the CART primarily functions within the nucleus accumbens (NAc) and ventral tegmental area, both of which are central to reward processing and the formation of addiction-related memories [57–59]. Increased CART expression during drug use can help inhibit excessive dopamine release, thereby preventing overactivation of the reward circuit and potentially reducing addictive behaviors [57]. For instance, injecting CART into the NAc can suppress the dopamine surge and behavioral responses triggered by cocaine use, contributing to a reduction in neural sensitization and addictive behaviors [57]. In our study, we observed a significant decrease in *Cartpt* expression following MA self-administration, which persisted throughout the subsequent MA12 and MA24 abstinence phases. This decrease in *Cartpt* expression may lead to a reduced level of the CART neuropeptide, which could increase sensitivity to the rewarding effects of drugs within the reward circuit, thereby enhancing the formation of drug-related memories. This heightened sensitization can reinforce addictive behaviors, potentially increasing the risk of drug dependence.

MA-induced changes in gene expression significantly affect the brain’s reward system and drug memory circuits, playing a crucial role in repeated drug use even after withdrawal. In this study, we identified ten key genes that showed the most significant changes in expression immediately after a short abstinence period following MA self-administration. These genes persist in the brain after drug cessation and are essential for restructuring the reward system and influencing the memory circuits related to drug use through alterations in neural plasticity. Gene expression can vary across the stages of abstinence, withdrawal, and relapse, depending on acute or chronic drug exposure. During the early withdrawal phase, the expression patterns of IEGs, such as *Fos*, *Egr1*, and *Nr4a1*, demonstrated a coordinated, activity-dependent transcriptional wave that initiates synaptic plasticity and ensemble formation [60, 61]. These patterns are consistent with frameworks in which CREB-centered transcription orchestrates striatal circuit remodeling during withdrawal and early abstinence [62, 63]. Mechanistically, the collective expression changes point to convergent regulation of striatal excitability and motivation; for example, 5-HT2C signaling and oxytocin-based modulation reduce stimulant self-administration or MA demand in preclinical models [64, 65]. Moreover, alterations in CART (*Cartpt*) implicate a peptide system that constrains dopaminergic reward, suggesting that reduced CART tone during early abstinence could remove a homeostatic brake on drug seeking [66]. Our study investigated gene expression patterns showing significant changes immediately after the MA reward and short-term abstinence, indicating their involvement in MA reward mechanisms. Taken together, these findings offer profound insights into the transcriptomic changes linked to MA dependence and highlight their functional significance. These findings enable the use of key transcriptomic markers of drug reward mechanisms to improve the accuracy and precision of diagnosing early-stage MA addiction.

## Limitation

This study has several limitations that should be considered when interpreting the findings. First, Bulk RNA-seq is well suited for comprehensively exploring transcriptome-wide changes in biological samples and is particularly useful for identifying key genes and monitoring overall transcriptomic dynamics over time, as in the present study. However, additional approaches such as single-cell or cell-type-specific sequencing would be valuable for examining expression changes in specific cell types in the brain with higher resolution.

Second, our behavioral model employed a short-access self-administration model that captures reward-related processes but does not encompass the full course of MA addiction. Extended-access paradigms that include cycles of drug taking, withdrawal, and relapse may better model pathological states across the addiction spectrum and should be pursued to test the generality of the present transcriptomic patterns.

Third, despite rigorous sample selection and RNA-seq quality control, the relatively small number of biological replicates per group reduces statistical power and may limit generalizability. Moreover, only male rats were studied; given increasing recognition of sex as a biological variable, the exclusion of females prevents assessment of sex-dependent transcriptomic trajectories.

Fourth, while our transcriptomic analyses identified several key genes, independent validation using approaches such as qPCR or Western blot may be required. Future studies incorporating such validations, along with functional assays, will be essential to strengthen mechanistic insights and to determine whether these genes play a causal role in reward-related neuroadaptations.

Finally, there are limitations in translating these findings to clinical contexts. Our analyses were conducted in rat striatal tissue; moving toward clinical application will require careful consideration of interspecies differences and prospective validation in large, independent human cohorts. In that context, the key genes described here represent candidates—rather than established biomarkers—that merit evaluation for detectability in accessible biofluids (e.g., peripheral blood), visualization via neuroimaging readouts, and correlation with behavioral measures relevant to MA use disorder.

## Supplementary Information

Below is the link to the electronic supplementary material.

Supplementary Material 1

Supplementary Material 2

Supplementary Material 3

Supplementary Material 4

Supplementary Material 5

Supplementary Material 6

Supplementary Material 7

Supplementary Material 8

Supplementary Material 9

Supplementary Material 10

Supplementary Material 11

## Data Availability

The datasets generated and/or analyzed during the current study are available in the Gene Expression Omnibus (GEO) database (accession numbers GSE211184 and GSE283226). All data generated or analyzed during this study are included in this published article and its supplementary information files.

## Abbreviations

ANOVA: Analysis of variance
ARGs: Addiction-related genes
BC: Betweenness centrality
CART: Cocaine- and amphetamine-regulated transcript
CC: Closeness centrality
CNS: Central nervous system
CTD: Comparative Toxicogenomics Database
DC: Degree centrality
DEGs: Differentially expressed genes
FC: Fold change
FDR: False discovery rate
FR-1: Fixed-ratio 1
IEGs: Immediate early genes
MA: Methamphetamine
MDT: Mediodorsal thalamus
PPI: Protein-protein interaction
QC: Quality control
rn6: Rat reference genome
SD: Sprague-Dawley
TMM: Trimmed Mean of M-values
