# Supplementary material for: Striatal transcriptomic alterations immediately after short-term abstinence from methamphetamine self-administration in rats
Source: Mol Brain. 2025 Nov 4;18:82. doi: 10.1186/s13041-025-01249-z (PMC12584396; doi:10.1186/s13041-025-01249-z)
Supplement: Supplementary file 6 — Supplementary Material 6 [file 13041_2025_1249_MOESM6_ESM.docx]

**Table S9.** Top 30 lists of BC, CC and DC genes in 317-PPI network

| **No.** | **EntrezID** | **Symbol** | **BC** | **FDR** | **EntrezID** | **Symbol** | **CC** | **FDR** | **EntrezID** | **Symbols** | **DC** | **FDR** |
| --- | --- | --- | --- | --- | --- | --- | --- | --- | --- | --- | --- | --- |
| 1 | 314322 | ***Fos*** | 0.247 | 0.000 | 314322 | ***Fos*** | 0.382 | 0.000 | 314322 | ***Fos*** | 60 | 0.000 |
| 2 | 24224 | ***Bcl2*** | 0.188 | 0.009 | 24224 | ***Bcl2*** | 0.368 | 0.009 | 24224 | ***Bcl2*** | 40 | 0.009 |
| 3 | 24329 | ***Egfr*** | 0.165 | 0.002 | 81646 | ***Creb1*** | 0.364 | 0.017 | 81646 | ***Creb1*** | 39 | 0.017 |
| 4 | 81646 | ***Creb1*** | 0.084 | 0.017 | 24329 | ***Egfr*** | 0.363 | 0.002 | 24329 | ***Egfr*** | 38 | 0.002 |
| 5 | 24179 | ***Agt*** | 0.078 | 0.000 | 29527 | ***Ptgs2*** | 0.352 | 0.000 | 24330 | ***Egr1*** | 33 | 0.000 |
| 6 | 24472 | ***Hspa1a*** | 0.076 | 0.000 | 25313 | ***Egf*** | 0.348 | 0.023 | 25313 | ***Egf*** | 32 | 0.023 |
| 7 | 29527 | ***Ptgs2*** | 0.073 | 0.000 | 24330 | ***Egr1*** | 0.347 | 0.000 | 29527 | ***Ptgs2*** | 31 | 0.000 |
| 8 | 297595 | ***P3h3*** | 0.063 | 0.000 | 24179 | ***Agt*** | 0.346 | 0.000 | 24179 | ***Agt*** | 30 | 0.000 |
| 9 | 25313 | ***Egf*** | 0.061 | 0.023 | 114856 | ***Dusp1*** | 0.339 | 0.000 | 25493 | ***Nfkbia*** | 25 | 0.015 |
| 10 | 315579 | ***Ubash3b*** | 0.058 | 0.020 | 25493 | ***Nfkbia*** | 0.339 | 0.015 | 79240 | ***Nr4a1*** | 25 | 0.000 |
| 11 | 311183 | ***Fnbp4*** | 0.056 | 0.003 | 25589 | ***Kdr*** | 0.337 | 0.000 | 114856 | ***Dusp1*** | 24 | 0.000 |
| 12 | 24409 | ***Grin2a*** | 0.051 | 0.001 | 24253 | ***Cebpb*** | 0.337 | 0.000 | 24253 | ***Cebpb*** | 22 | 0.000 |
| 13 | 297942 | ***Pnisr*** | 0.050 | 0.000 | 24323 | ***Edn1*** | 0.334 | 0.001 | 24323 | ***Edn1*** | 20 | 0.001 |
| 14 | 25589 | ***Kdr*** | 0.048 | 0.000 | 310553 | ***Tlr2*** | 0.330 | 0.000 | 114090 | ***Egr2*** | 20 | 0.000 |
| 15 | 114856 | ***Dusp1*** | 0.047 | 0.000 | 114851 | ***Cdkn1a*** | 0.327 | 0.000 | 100360880 | ***Fosb*** | 20 | 0.000 |
| 16 | 171071 | ***Ppp1r15a*** | 0.046 | 0.002 | 25491 | ***Nes*** | 0.324 | 0.009 | 24517 | ***Junb*** | 20 | 0.000 |
| 17 | 304832 | ***Cdc73*** | 0.045 | 0.002 | 79240 | ***Nr4a1*** | 0.321 | 0.000 | 25589 | ***Kdr*** | 19 | 0.000 |
| 18 | 292793 | ***Sbsn*** | 0.044 | 0.017 | 24471 | ***Hspb1*** | 0.321 | 0.000 | 24409 | ***Grin2a*** | 17 | 0.001 |
| 19 | 24253 | ***Cebpb*** | 0.042 | 0.000 | 24472 | ***Hspa1a*** | 0.319 | 0.000 | 310553 | ***Tlr2*** | 16 | 0.000 |
| 20 | 114090 | ***Egr2*** | 0.040 | 0.000 | 114090 | ***Egr2*** | 0.319 | 0.000 | 25504 | ***Oxt*** | 16 | 0.000 |
| 21 | 24716 | ***Ret*** | 0.040 | 0.038 | 63879 | ***Xiap*** | 0.318 | 0.003 | 266734 | ***Npas4*** | 16 | 0.005 |
| 22 | 296368 | ***Ube2c*** | 0.040 | 0.039 | 25504 | ***Oxt*** | 0.311 | 0.000 | 58853 | ***Nr4a3*** | 15 | 0.000 |
| 23 | 308097 | ***Tagap*** | 0.037 | 0.011 | 58853 | ***Nr4a3*** | 0.310 | 0.000 | 29707 | ***Gabra5*** | 15 | 0.006 |
| 24 | 25062 | ***Gpd2*** | 0.035 | 0.002 | 170496 | ***Lcn2*** | 0.308 | 0.006 | 114851 | ***Cdkn1a*** | 14 | 0.000 |
| 25 | 63879 | ***Xiap*** | 0.034 | 0.003 | 25620 | ***Crem*** | 0.308 | 0.000 | 25491 | ***Nes*** | 14 | 0.009 |
| 26 | 24539 | ***Lpl*** | 0.034 | 0.021 | 29376 | ***Irs2*** | 0.308 | 0.000 | 24471 | ***Hspb1*** | 14 | 0.000 |
| 27 | 170496 | ***Lcn2*** | 0.033 | 0.006 | 100360880 | ***Fosb*** | 0.307 | 0.000 | 63879 | ***Xiap*** | 13 | 0.003 |
| 28 | 25248 | ***Cnr1*** | 0.031 | 0.000 | 24409 | ***Grin2a*** | 0.307 | 0.001 | 25635 | ***Mc4r*** | 13 | 0.015 |
| 29 | 304268 | ***Rasl11a*** | 0.031 | 0.000 | 29175 | ***Ctsk*** | 0.306 | 0.024 | 25695 | ***Cebpd*** | 13 | 0.001 |
| 30 | 81670 | ***Gpt*** | 0.031 | 0.001 | 25635 | ***Mc4r*** | 0.305 | 0.015 | 25187 | ***Htr2c*** | 13 | 0.000 |
