# Supplementary material for: Striatal transcriptomic alterations immediately after short-term abstinence from methamphetamine self-administration in rats
Source: Mol Brain. 2025 Nov 4;18:82. doi: 10.1186/s13041-025-01249-z (PMC12584396; doi:10.1186/s13041-025-01249-z)
Supplement: Supplementary file 7 — Supplementary Material 7 [file 13041_2025_1249_MOESM7_ESM.docx]

| **No.** | **EntrezID** | ***Symbols*** | **ARG** | **BC** | **CC** | **DC** | **Name** | **FDR** |
| --- | --- | --- | --- | --- | --- | --- | --- | --- |
| 1 | 312382 | ***Abcg2*** |  | 0.013 | 0.292 | 7 | ATP binding cassette subfamily G member 2 | 0.005 |
| 2 | 81633 | ***Acta2*** |  | 0.015 | 0.305 | 12 | actin alpha 2, smooth muscle | 0.000 |
| 3 | 24179 | ***Agt*** | ● | 0.078 | 0.346 | 30 | angiotensinogen | 0.000 |
| 4 | 293524 | ***Bag3*** |  | 0.025 | 0.280 | 9 | BAG cochaperone 3 | 0.000 |
| 5 | 24224 | ***Bcl2*** | ● | 0.188 | 0.368 | 40 | BCL2, apoptosis regulator | 0.009 |
| 6 | 29131 | ***Cartpt*** | ● | 0.025 | 0.249 | 6 | CART prepropeptide | 0.000 |
| 7 | 24253 | ***Cebpb*** | ● | 0.042 | 0.337 | 22 | CCAAT/enhancer binding protein beta | 0.000 |
| 8 | 25695 | ***Cebpd*** | ● | 0.025 | 0.303 | 13 | CCAAT/enhancer binding protein delta | 0.001 |
| 9 | 81646 | ***Creb1*** | ● | 0.084 | 0.364 | 39 | cAMP responsive element binding protein 1 | 0.017 |
| 10 | 114856 | ***Dusp1*** |  | 0.047 | 0.339 | 24 | dual specificity phosphatase 1 | 0.000 |
| 11 | 24323 | ***Edn1*** |  | 0.014 | 0.334 | 20 | endothelin 1 | 0.001 |
| 12 | 25313 | ***Egf*** |  | 0.061 | 0.348 | 32 | epidermal growth factor | 0.023 |
| 13 | 24329 | ***Egfr*** | ● | 0.165 | 0.363 | 38 | epidermal growth factor receptor | 0.002 |
| 14 | 24330 | ***Egr1*** | ● | 0.028 | 0.347 | 33 | early growth response 1 | 0.000 |
| 15 | 114090 | ***Egr2*** | ● | 0.040 | 0.319 | 20 | early growth response 2 | 0.000 |
| 16 | 59323 | ***Erbb4*** |  | 0.025 | 0.302 | 8 | erb-b2 receptor tyrosine kinase 4 | 0.018 |
| 17 | 314322 | ***Fos*** | ● | 0.247 | 0.382 | 60 | Fos proto-oncogene, AP-1 transcription factor subunit | 0.000 |
| 18 | 29707 | ***Gabra5*** |  | 0.023 | 0.254 | 15 | gamma-aminobutyric acid type A receptor alpha 5 subunit | 0.006 |
| 19 | 291005 | ***Gadd45g*** | ● | 0.013 | 0.291 | 9 | growth arrest and DNA-damage-inducible, gamma | 0.000 |
| 20 | 60449 | ***Gnb3*** |  | 0.025 | 0.266 | 13 | G protein subunit beta 3 | 0.020 |
| 21 | 294962 | ***Gnb4*** |  | 0.016 | 0.259 | 12 | G protein subunit beta 4 | 0.049 |
| 22 | 24409 | ***Grin2a*** | ● | 0.051 | 0.307 | 17 | glutamate ionotropic receptor NMDA type subunit 2A | 0.001 |
| 23 | 24472 | ***Hspa1a*** | ● | 0.076 | 0.319 | 12 | heat shock 70kD protein 1A | 0.000 |
| 24 | 25187 | ***Htr2c*** | ● | 0.024 | 0.300 | 13 | 5-hydroxytryptamine receptor 2C | 0.000 |
| 25 | 292892 | ***Irf3*** |  | 0.027 | 0.290 | 11 | interferon regulatory factor 3 | 0.000 |
| 26 | 29376 | ***Irs2*** |  | 0.016 | 0.308 | 9 | insulin receptor substrate 2 | 0.000 |
| 27 | 24517 | ***Junb*** | ● | 0.021 | 0.304 | 20 | JunB proto-oncogene, AP-1 transcription factor subunit | 0.000 |
| 28 | 25743 | ***Kcnj6*** |  | 0.017 | 0.268 | 9 | potassium inwardly-rectifying channel, subfamily J, member 6 | 0.015 |
| 29 | 363630 | ***Kdm6b*** |  | 0.025 | 0.259 | 6 | lysine demethylase 6B | 0.000 |
| 30 | 25589 | ***Kdr*** |  | 0.048 | 0.337 | 19 | kinase insert domain receptor | 0.000 |
| 31 | 84410 | ***Klf5*** |  | 0.013 | 0.271 | 8 | Kruppel-like factor 5 | 0.000 |
| 32 | 170496 | ***Lcn2*** |  | 0.033 | 0.308 | 11 | lipocalin 2 | 0.006 |
| 33 | 24539 | ***Lpl*** |  | 0.034 | 0.265 | 8 | lipoprotein lipase | 0.021 |
| 34 | 25635 | ***Mc4r*** | ● | 0.029 | 0.305 | 13 | melanocortin 4 receptor | 0.015 |
| 35 | 25491 | ***Nes*** |  | 0.016 | 0.324 | 14 | nestin | 0.009 |
| 36 | 114519 | ***Nfil3*** |  | 0.024 | 0.295 | 12 | nuclear factor, interleukin 3 regulated | 0.000 |
| 37 | 25493 | ***Nfkbia*** | ● | 0.029 | 0.339 | 25 | NFKB inhibitor alpha | 0.015 |
| 38 | 266734 | ***Npas4*** |  | 0.017 | 0.295 | 16 | neuronal PAS domain protein 4 | 0.005 |
| 39 | 79240 | ***Nr4a1*** | ● | 0.014 | 0.321 | 25 | nuclear receptor subfamily 4, group A, member 1 | 0.000 |
| 40 | 25504 | ***Oxt*** | ● | 0.023 | 0.311 | 16 | oxytocin/neurophysin I prepropeptide | 0.000 |
| 41 | 297595 | ***P3h3*** |  | 0.063 | 0.275 | 6 | prolyl 3-hydroxylase 3 | 0.000 |
| 42 | 171071 | ***Ppp1r15a*** |  | 0.046 | 0.269 | 6 | protein phosphatase 1, regulatory subunit 15A | 0.002 |
| 43 | 29527 | ***Ptgs2*** |  | 0.073 | 0.352 | 31 | prostaglandin-endoperoxide synthase 2 | 0.000 |
| 44 | 24716 | ***Ret*** |  | 0.040 | 0.282 | 7 | ret proto-oncogene | 0.038 |
| 45 | 81574 | ***Scn1a*** |  | 0.013 | 0.249 | 11 | sodium voltage-gated channel alpha subunit 1 | 0.001 |
| 46 | 116638 | ***Slc17a7*** |  | 0.023 | 0.293 | 10 | solute carrier family 17 member 7 | 0.007 |
| 47 | 310553 | ***Tlr2*** |  | 0.018 | 0.330 | 16 | toll-like receptor 2 | 0.000 |
| 48 | 296368 | ***Ube2c*** |  | 0.040 | 0.287 | 6 | ubiquitin-conjugating enzyme E2C | 0.039 |
| 49 | 63879 | ***Xiap*** |  | 0.034 | 0.318 | 13 | X-linked inhibitor of apoptosis | 0.003 |
| 50 | 353227 | ***Zbtb16*** | ● | 0.026 | 0.246 | 7 | zinc finger and BTB domain containing 16 | 0.027 |

**Table S10.** List of 50 hub genes in 317-PPI network
