# Supplementary material for: Striatal transcriptomic alterations immediately after short-term abstinence from methamphetamine self-administration in rats
Source: Mol Brain. 2025 Nov 4;18:82. doi: 10.1186/s13041-025-01249-z (PMC12584396; doi:10.1186/s13041-025-01249-z)
Supplement: Supplementary file 8 — Supplementary Material 8 [file 13041_2025_1249_MOESM8_ESM.docx]

| **No.** | **EntrezID** | **Symbols** | **Name** | **FDR** | **No.** | **EntrezID** | **Symbols** | **Name** | **FDR** |
| --- | --- | --- | --- | --- | --- | --- | --- | --- | --- |
| 1 | 25026 | *Adm* | adrenomedullin | 0.006 | 31 | 24409 | *Grin2a* | glutamate ionotropic receptor NMDA type subunit 2A | 0.001 |
| 2 | 24179 | *Agt* | angiotensinogen | 0.000 | 32 | 29430 | *Hap1* | huntingtin-associated protein 1 | 0.046 |
| 3 | 54323 | *Arc* | activity-regulated cytoskeleton-associated protein | 0.000 | 33 | 24472 | *Hspa1a* | heat shock 70kD protein 1A | 0.000 |
| 4 | 65036 | *Bche* | butyrylcholinesterase | 0.039 | 34 | 24471 | *Hspb1* | heat shock protein family B (small) member 1 | 0.000 |
| 5 | 24224 | *Bcl2* | BCL2, apoptosis regulator | 0.009 | 35 | 25187 | *Htr2c* | 5-hydroxytryptamine receptor 2C | 0.000 |
| 6 | 29131 | *Cartpt* | CART prepropeptide | 0.000 | 36 | 361673 | *Ifitm3* | interferon induced transmembrane protein 3 | 0.015 |
| 7 | 114851 | *Cdkn1a* | cyclin-dependent kinase inhibitor 1A | 0.000 | 37 | 24517 | *Junb* | JunB proto-oncogene, AP-1 transcription factor subunit | 0.000 |
| 8 | 24253 | *Cebpb* | CCAAT/enhancer binding protein beta | 0.000 | 38 | 81813 | *Klf10* | Kruppel-like factor 10 | 0.000 |
| 9 | 25695 | *Cebpd* | CCAAT/enhancer binding protein delta | 0.001 | 39 | 361506 | *Leng8* | leukocyte receptor cluster member 8 | 0.000 |
| 10 | 299314 | *Cfp* | complement factor properdin | 0.023 | 40 | 366960 | *Maff* | MAF bZIP transcription factor F | 0.002 |
| 11 | 301434 | *Clk1* | CDC-like kinase 1 | 0.009 | 41 | 25635 | *Mc4r* | melanocortin 4 receptor | 0.015 |
| 12 | 25248 | *Cnr1* | cannabinoid receptor 1 | 0.000 | 42 | 361508 | *Mzf1* | myeloid zinc finger 1 | 0.000 |
| 13 | 81646 | *Creb1* | cAMP responsive element binding protein 1 | 0.017 | 43 | 29227 | *Nfib* | nuclear factor I/B | 0.000 |
| 14 | 25620 | *Crem* | cAMP responsive element modulator | 0.000 | 44 | 25493 | *Nfkbia* | NFKB inhibitor alpha | 0.015 |
| 15 | 25420 | *Cryab* | crystallin, alpha B | 0.005 | 45 | 288475 | *Nptx2* | neuronal pentraxin 2 | 0.000 |
| 16 | 114587 | *Dlk1* | delta like non-canonical Notch ligand 1 | 0.046 | 46 | 79240 | *Nr4a1* | nuclear receptor subfamily 4, group A, member 1 | 0.000 |
| 17 | 60587 | *Dusp4* | dual specificity phosphatase 4 | 0.000 | 47 | 58853 | *Nr4a3* | nuclear receptor subfamily 4, group A, member 3 | 0.000 |
| 18 | 171109 | *Dusp5* | dual specificity phosphatase 5 | 0.000 | 48 | 25504 | *Oxt* | oxytocin/neurophysin I prepropeptide | 0.000 |
| 19 | 116663 | *Dusp6* | dual specificity phosphatase 6 | 0.018 | 49 | 29433 | *Pak3* | p21 (RAC1) activated kinase 3 | 0.003 |
| 20 | 24329 | *Egfr* | epidermal growth factor receptor | 0.002 | 50 | 287422 | *Per1* | period circadian regulator 1 | 0.000 |
| 21 | 24330 | *Egr1* | early growth response 1 | 0.000 | 51 | 63840 | *Per2* | period circadian regulator 2 | 0.000 |
| 22 | 114090 | *Egr2* | early growth response 2 | 0.000 | 52 | 360814 | *Rasal1* | RAS protein activator like 1 | 0.049 |
| 23 | 25129 | *Egr4* | early growth response 4 | 0.000 | 53 | 29481 | *Rgs9* | regulator of G-protein signaling 9 | 0.000 |
| 24 | 290639 | *Fcho1* | FCH and mu domain containing endocytic adaptor 1 | 0.000 | 54 | 361696 | *Rps6kb2* | ribosomal protein S6 kinase B2 | 0.000 |
| 25 | 314322 | *Fos* | Fos proto-oncogene, AP-1 transcription factor subunit | 0.000 | 55 | 84402 | *Sfrp1* | secreted frizzled-related protein 1 | 0.013 |
| 26 | 100360880 | *Fosb* | FosB proto-oncogene, AP-1 transcription factor subunit | 0.000 | 56 | 29482 | *Slc1a2* | solute carrier family 1 member 2 | 0.006 |
| 27 | 25451 | *Gabrb2* | gamma-aminobutyric acid type A receptor beta 2 subunit | 0.036 | 57 | 54305 | *Sstr2* | somatostatin receptor 2 | 0.000 |
| 28 | 291005 | *Gadd45g* | growth arrest and DNA-damage-inducible, gamma | 0.000 | 58 | 499016 | *Tulp4* | TUB like protein 4 | 0.001 |
| 29 | 83585 | *Gda* | guanine deaminase | 0.026 | 59 | 24877 | *Vsnl1* | visinin-like 1 | 0.008 |
| 30 | 25062 | *Gpd2* | glycerol-3-phosphate dehydrogenase 2 | 0.002 | 60 | 353227 | *Zbtb16* | zinc finger and BTB domain containing 16 | 0.027 |

Table S11. List of 60 addiction-related genes in 317-PPI network
